# In Vivo and in Vitro activity of colistin-conjugated bimetallic silver-copper oxide nanoparticles against Pandrug-resistant *Pseudomonas aeruginosa*

**DOI:** 10.1186/s12866-024-03358-6

**Published:** 2024-06-17

**Authors:** Asmaa Abdul Hak, Hamdallah H. Zedan, Hadir A. El-Mahallawy, Gharieb S. El-Sayyad, Mai M. Zafer

**Affiliations:** 1 Department of Microbiology and Immunology, Faculty of Pharmacy, Ahram Canadian University, Giza, Egypt; 2 Department of Microbiology and Immunology, Faculty of Pharmacy, Cairo University, Cairo, Egypt; 3 Department of Clinical Pathology, National Cancer Institute, Cairo University, Cairo, Egypt; 4 Department of Microbiology and Immunology, Faculty of Pharmacy, Galala University, New Galala City, Suez Egypt; 5 Drug Microbiology Lab, Drug Radiation Research Department, National Center for Radiation Research and Technology (NCRRT), Egyptian Atomic Energy Authority (EAEA), Cairo, Egypt

**Keywords:** Bimetallic nanoparticles, Antimicrobial activity, *Pseudomonas aeruginosa*, Antimicrobial resistance, Virulence factors

## Abstract

**Background:**

Addressing microbial resistance urgently calls for alternative treatment options. This study investigates the impact of a bimetallic formulation containing colistin, silver, and copper oxide on a pandrug-resistant, highly virulent *Pseudomonas aeruginosa (P. aeruginosa)* isolate from a cancer patient at the National Cancer Institute, Cairo University, Egypt.

**Methods:**

Silver nanoparticles (Ag NPs), copper oxide nanoparticles (CuO NPs), and bimetallic silver-copper oxide nanoparticles (Ag-CuO NPs) were synthesized using gamma rays, combined with colistin (Col), and characterized by various analytical methods. The antimicrobial activity of Col-Ag NPs, Col-CuO NPs, and bimetallic Col-Ag-CuO NPs against *P. aeruginosa* was evaluated using the agar well diffusion method, and their minimum inhibitory concentration (MIC) was determined using broth microdilution. Virulence factors such as pyocyanin production, swarming motility, and biofilm formation were assessed before and after treatment with bimetallic Col-Ag-CuO NPs. The in vivo efficacy was evaluated using the *Galleria mellonella* model, and antibacterial mechanism were examined through membrane leakage assay.

**Results:**

The optimal synthesis of Ag NPs occurred at a gamma ray dose of 15.0 kGy, with the highest optical density (OD) of 2.4 at 375 nm. Similarly, CuO NPs had an optimal dose of 15.0 kGy, with an OD of 1.5 at 330 nm. Bimetallic Ag-CuO NPs were most potent at 15.0 kGy, yielding an OD of 1.9 at 425 nm. The MIC of colistin was significantly reduced when combined with nanoparticles: 8 µg/mL for colistin alone, 0.046 µg/mL for Col-Ag NPs, and 0.0117 µg/mL for Col-Ag-CuO NPs. Bimetallic Col-Ag-CuO NPs reduced the MIC four-fold compared to Col-Ag NPs. Increasing the sub-inhibitory concentration of bimetallic nanoparticles from 0.29 × 10^-2^ to 0.58 × 10^-2^ µg/mL reduced *P. aeruginosa* swarming by 32–64% and twitching motility by 34–97%. At these concentrations, pyocyanin production decreased by 39–58%, and biofilm formation was inhibited by 33–48%. The nanoparticles were non-toxic to *Galleria mellonella*, showing 100% survival by day 3, similar to the saline-treated group.

**Conclusions:**

The synthesis of bimetallic Ag-CuO NPs conjugated with colistin presents a promising alternative treatment for combating the challenging *P. aeruginosa* pathogen in hospital settings. Further research is needed to explore and elucidate the mechanisms underlying the inhibitory effects of colistin-bimetallic Ag-CuO NPs on microbial persistence and dissemination.

## Introduction

*Pseudomonas aeruginosa* poses a crucial threat as an opportunistic pathogen in hospital environments [1]. Recognizing its exceptional resistance profile, the World Health Organization categorized it as critically prioritized for the development of new antibiotics [2].

The adaptability of *P. aeruginosa* genome, coupled with its biofilm-forming proficiency and array of virulence factors, underscores its pathogenicity, particularly in immunocompromised cancer patients, leading to elevated mortality rates [3]. Antimicrobial resistance, a global challenge, complicates treatment options, particularly in countries like Egypt [4, 5].

Reports of *P. aeruginosa* strains resistant even to colistin have surfaced, amplifying public health concerns [6, 7]. This phenomenon is exacerbated by the overuse and misuse of antibiotics, coupled with socioeconomic factors such as inadequate hygiene practices and lax infection control measures [8–10]. Infections caused by pandrug-resistant *P. aeruginosa* not only heighten mortality rates but also disrupt anticancer therapies [11]. Therefore, there is an urgent need for innovative antimicrobial agents effective against this resilient pathogen. Nanoparticles (NPs) have emerged as a promising strategy to combat multidrug-resistant bacterial infections [12, 13]. Metal-based nanoparticles, in particular, exhibit antibacterial properties, primarily through mechanisms involving reactive oxygen species and bacterial membrane disruption [14, 15]. Combining antibiotics with metal nanoparticles enhances antibacterial efficacy, with the increased surface area of nanoparticles facilitating bacterial cell damage [16].

The unique properties of bimetallic nanomaterials, stemming from synergistic effects between different metallic components, have gained significant interest [17–19]. Notably, bimetallic silver-copper oxide nanoparticles (Ag-CuO NPs) have demonstrated versatile plasmonic properties, making them attractive for various applications, including sensors and catalysis [20].

In this study, we investigated the antibacterial and anti-virulence potential of the synthesized mono- and bimetallic formulations of colistin-conjugated silver and copper oxide nanoparticles against pandrug-resistant and highly virulent clinical isolate of *P. aeruginosa*, obtained from immunocompromised hospitalized pediatric cancer patient.

## Materials and methods

### Chemicals and reagents

The production of NPs was applied using analytical-grade chemicals such as Ag NO_3_ and CuSO_4_. 5H_2_O (Sigma Aldrich, UK). Muller-Hinton agar and broth, Tryptic Soy Broth (TSB) used for microbiological testing were obtained from Oxoid, UK.

### Gamma radiation

Processes for gamma irradiation were carried out at the National Center for Radiation Research and Technology (NCRRT) in Cairo, Egypt. The samples were gamma-irradiated in solution form using ^60^Co-Gamma chamber 4000-A-India as the radiation source, with the radiation time being calculated to be 0.843 kGy per hour (dose rate).

### Synthesis of Col-Ag NPs, Col-CuO NPs and bimetallic Col-Ag-CuO NPs

Ag NPs, CuO NPs, and bimetallic Ag-CuO NPs were synthesized using gamma rays (as a both direct and indirect reducing tool). Because powerful reducing electrons, known as e^-^_aq_, were freed by gamma rays in aqueous solutions, metal ions were subsequently reduced. This process is known as direct reduction. While the radiolysis byproducts H^•^ and OH^•^ interact, and reduce metal ions, the indirect reduction was caused by H^•^ and OH^•^ [13].

Several solution samples (50 mL) containing (1.0 mM) Ag NO_3_ were combined with colistin sulphate solution to create Ag NPs. Similarly, numerous solution (50 mL) of (1.0 mM) CuSO_4_ .5H_2_O were combined with colistin sulphate solution to synthesize CuO NPs. Multiple solution samples containing (1.0 mM; 25 mL) Ag NO_3_ and (1.0 mM; 25 mL) CuSO_4_. 5H_2_O were combined with colistin sulphate solution to synthesize bimetallic Ag-CuO NPs.

Prior to gamma radiation, the pH of each sample was determined and adjusted to neutral (pH 7). Then, fixed dosages of gamma radiation (15 kGy) were applied to the solutions at room temperature. The optical density (OD.) of the generated NPs at a particular and defined wavelength was measured using UV-Vis. spectra. In the case of bimetallic Ag-CuO NPs, the relative Ag/CuO concentration was studied as it is a major determinant in addition to the dosage [21, 22].

### Characterization of the synthesized NPs

Using a UV-Vis. spectrophotometer (JASCO V-560) at certain wavelengths, the absorbance and optical properties of produced Col-Ag NPs, Col-CuO NPs, and bimetallic Col-Ag-CuO NPs were examined. For Auto-zero reasons, a sample devoid of any metal ions was additionally included. All samples were initially screened for optical characteristics and to establish the fixed wavelengths used to calculate absorbance. To ascertain the mean size distribution of the produced NPs, measurements of dynamic light scattering were made at the St. Barbara, California, USA facility using the DLS-PSS-NICOMP 380-ZLS particles sized system. 100 µL of NPs specimens were transferred to a temporary, tiny cuvette. Five procedures were carried out after equilibration at a temperature of 25 ± 2 °C for 2.0 min. In addition, the form, appearance, and average particle size of the produced NPs were investigated using a high-resolution transmission electron microscope (HR-TEM, JEM2100, Jeol, Japan). NPs samples used for TEM studies were drop-coated into carbon-coated TEM grids following dried in an incubator at 37.0 ± 2 °C. Additionally, using the XRD-6000 lists, Shimadzu equipment, SSI, Japan, it was possible to assess the crystallization, crystallite size, and/or structure of the generated NPs. The magnitude of the diffracted X-rays was measured using the diffracted angle 2θ. To clarify surface shape, border size, and the distribution of the generated NPs surrounding GA, an analysis using SEM (SEM, ZEISS, EVO-MA10, Germany) was performed.

### Bacterial strain

The study was conducted using a clinical isolate of *P. aeruginosa* obtained from the blood culture of a pediatric cancer patient hospitalized at the National Cancer Institute, Cairo University, Egypt, in September 2022. The *P. aeruginosa* isolate was initially identified using standard microbiological techniques such as gram staining and oxidase testing. Further confirmation was conducted using the Vitek® 2 automated system (bioMérieux, Marcyl’Etoile, France) in the microbiology laboratory at the National Cancer Institute. This isolate was selected from a collection of isolates for its high virulence and susceptibility profile. It exhibited strong biofilm production, pyocyanin production, and demonstrated lethality in the *Galleria mellonella* model.

### Antimicrobial susceptibility testing

Antimicrobial susceptibility testing was conducted using VITEK 2 automated machine (bioMérieux, Marcy l’Etoile, France) against a panel of antibiotics including ceftazidime, cefazolin, cefuroxime, ceftriaxone, cefepime, ampicillin, amoxicillin-clavulanate, piperacillin-tazobactam, ceftolozane-tazobactam, amikacin, gentamicin, ertapenem, imipenem, meropenem, trimethoprim-sulfamethoxazole, nitrofurantoin, ciprofloxacin, levofloxacin, and tigecycline. Interpretation of results was based on CLSI guidelines [23]. Colistin susceptibility was assessed using the broth microdilution method and interpreted according to EUCAST guidelines [24].

### In-vitro Antimicrobial activity of Col-AgNPs and Col-Ag-CuONPs

The antimicrobial activity of Col-Ag NPs, Col-CuO NPs, and Col-Ag-CuO NPs composites was assessed using the agar well diffusion method [25]. A suspension of *P. aeruginosa* isolate at a concentration of 1 × 10^8^ CFU/mL was prepared and streaked on Muller-Hinton agar plates (Oxoid, UK). Three concentrations (100%, 50%, and 25%) of Col-Ag NPs, Col-CuO NPs, and Col-Ag-CuO NPs were tested, with 50 µL of each concentration added to the wells. Incubation of the plates at 37 °C for 24 h allowed evaluation of antimicrobial activity by measuring the diameters of the zones of inhibition. The experiments were conducted in triplicate, and average results were calculated.

For the determination of minimum inhibitory concentration, the least effective concentration of Col-Ag NPs, Col-CuO NPs, and Col-Ag-CuO NPs observed in the agar well diffusion assay was utilized to generate a wide range of serial dilutions in a 96-well plate using cation-adjusted Muller–Hinton (CAMH) broth. The concentration of colistin within this range was 25 µg/mL, with concentrations spanning from 25 to 3 × 10^− 6^ µg/mL in the 96-well plate. A cell suspension was prepared by suspending 3 colonies of overnight growth in saline to achieve a density of 1 × 10^8^ CFU/mL. This suspension was further diluted to 5 × 10^6^ CFU/mL using cation-adjusted Muller–Hinton (CAMH) broth. Subsequently, 10 µL of the inoculum was transferred to the wells containing 100 µL of the diluted preparations to attain a final inoculum size of 5 × 10^5 CFU/mL. The plates were then incubated at 37 °C for 24 h [26].

### Anti-virulence activity

#### Motility test

The impact of the bimetallic Col-Ag-CuO NPs alloy on *P. aeruginosa* swarming and twitching motilities was investigated following the method outlined by Luo et al. [25]. An overnight culture of *P. aeruginosa* grown in Luria Bertani broth (LB) was adjusted to reach an OD_600_ = 1. The swarming medium was prepared by supplementing LB with 0.5% (w/v) casamino acids and 0.4% (w/v) Bacto agar, while the twitching medium consisted of 1.5% LB agar. Two sub-inhibitory concentrations of the Col-Ag-CuO NPs alloy were added to the agar media prior to solidification (Concentration 1 = 0.29 × 10^− 2^ µg/mL, Concentration 2 = 0.58 × 10^− 2^ µg/mL), and then 2.5 µL of the inoculum was applied to the swarming and twitching agar plates, followed by incubation at 35 °C for 24 h. The migration zones appearing on the agar plates were monitored. All motility experiments were conducted in triplicate.

### Pyocyanin production

The pyocyanin production assay was conducted following the protocol outlined by Wang et al. [27]. Briefly, a culture of *P. aeruginosa* isolate was grown in P*seudomonas* broth supplemented with two sub-inhibitory concentrations of Col-Ag-CuO NPs alloy (Concentration 1 = 0.29 × 10^− 2^ µg/mL, Concentration 2 = 0.58 × 10^− 2^ µg/mL) at 37 °C for 24 h. The culture was then centrifuged, and the supernatant collected. Subsequently, the supernatant was mixed with chloroform by vortexing and centrifuged at 8000 rpm for 10 min. The resulting mixture was then combined with 0.2 M HCl in a new centrifuge tube and centrifuged again. The organic layer was removed, and the absorbance of pyocyanin was measured at 520 nm.

### Biofilm formation

Overnight cell growth in Tryptic Soy Broth (TSB) was diluted (1:100) and added to a 96-well microtiter plate, with one set of wells serving as a control and the others treated with two sub-inhibitory concentrations of the nanocomposite solution (Conc. 1 = 0.29 × 10^− 2^ µg/mL, Conc. 2 = 0.58 × 10^− 2^ µg/mL). The plate was then incubated at 35 °C for 24 h without agitation. Planktonic cells were removed, and the attached cells were washed with phosphate buffer solution (PBS), air dried for 15 min, and stained with 0.1% crystal violet for 30 min at room temperature. After washing off the dye, the stained biofilm was dissolved using 33% glacial acetic acid, and the OD was measured at a wavelength of 570 nm [28].

The same procedure was repeated to evaluate the effect of the nanocomposites on mature pre-formed biofilms of *P. aeruginosa*. In this case, overnight bacterial cultures were allowed to form biofilms in the microtiter plate for 24 h at 35 °C without agitation. After removing planktonic cells, fresh TSB media containing different concentrations of the alloy were added to the wells with attached cells and incubated for an additional 24 h at 35 °C [29]. Biofilm disruption ability was determined by measuring the OD at 570 nm after staining with crystal violet dye. Each test was performed in triplicate, and the average value was calculated. Furthermore, another set of cultures, not stained with crystal violet, underwent viable count analysis. Attached cells were scratched with a micropipette tip and dispersed in saline using dilutions up to 10^− 5^ to assess the survival ability of biofilm cells in the presence of the nanocomposite in both scenarios [30].

### In vivo activity using *Galleria mellonella model*

The method employed in this study was implemented according to Thomaz et al. [31]. *G. mellonella* larvae weighing between 150 and 220 mg, obtained in the final (7th ) larval instar, were selected for experimentation. Only larvae exhibiting robust movement and free of myelinization were utilized. Five groups, each consisting of 10 *G. mellonella* larvae, were monitored for a duration of 72 h. The experimental groups were configured as follows:

1. Positive control: Larvae were injected with 10 µL of overnight culture of the *P. aeruginosa* clinical isolate, adjusted in saline to reach a concentration of 10^4^ CFU/mL.
2. Toxicity assessment: Larvae were injected with 10 µL of the higher concentration of the nanocomposite (Conc. 2 = 0.58 × 10^− 2^ µg/mL).
3. Treatment groups: Larvae were treated with either concentration 1 (0.29 × 10^− 2^ µg/mL) or concentration 2 (0.58 × 10^− 2^ µg/mL) of the nanocomposite. After a 2-hour incubation at 28 °C, these groups were infected with 10 µL of the bacterial strain, similar to the positive control group.
4. Negative control: Larvae were injected with 10 µL of saline.

Injection was administered into the haemocoel of the last right pro-leg using a 10 µL Hamilton micro syringe. The larvae were then placed in Petri dishes and incubated at 37 °C, with monitoring conducted over the course of 72 h. Survival curves were constructed based on the criteria of live or dead observations.

### Effect of the synthesized NPs on protein leakage from *P. aeruginosa* cell membranes

Pure 18 h *P. aeruginosa* culture was set at 0.5 McFarland (1 × 10^8^ CFU/mL) and 100 µL was injected into 10 mL of the nutrient broth containing well-sonicated and dispersed Col-Ag NPs, Col-CuO NPs, and Col-Ag-CuO NPs at various concentrations (0.125, 0.25, 0.5, and 1.0 mg/mL). Nanocomposites-free broth injected with culture had been used as the control. All the treated samples were incubated at 37 °C for 5 h. and then centrifuged for 15 min. at 5000 rpm [32]. For the different samples, 100 µL supernatant was combined with 1 mL of Bradford reagent. Optical density was then measured at 595 nm in the next 10 min of dark incubation [32].

### Statistical analysis

ONE WAY ANOVA at P 0.05, the least significant difference (LSD) summary, and Duncan’s multiple regions were used to statistically analyze the obtained results [33]. Using SPSS software (version 15), analyses and evaluations of the data and findings were conducted.

## Results and discussion

### Synthesis of Col-Ag NPs, Col-CuO NPs and Col-Ag-CuO NPs

**Fig. 1:**
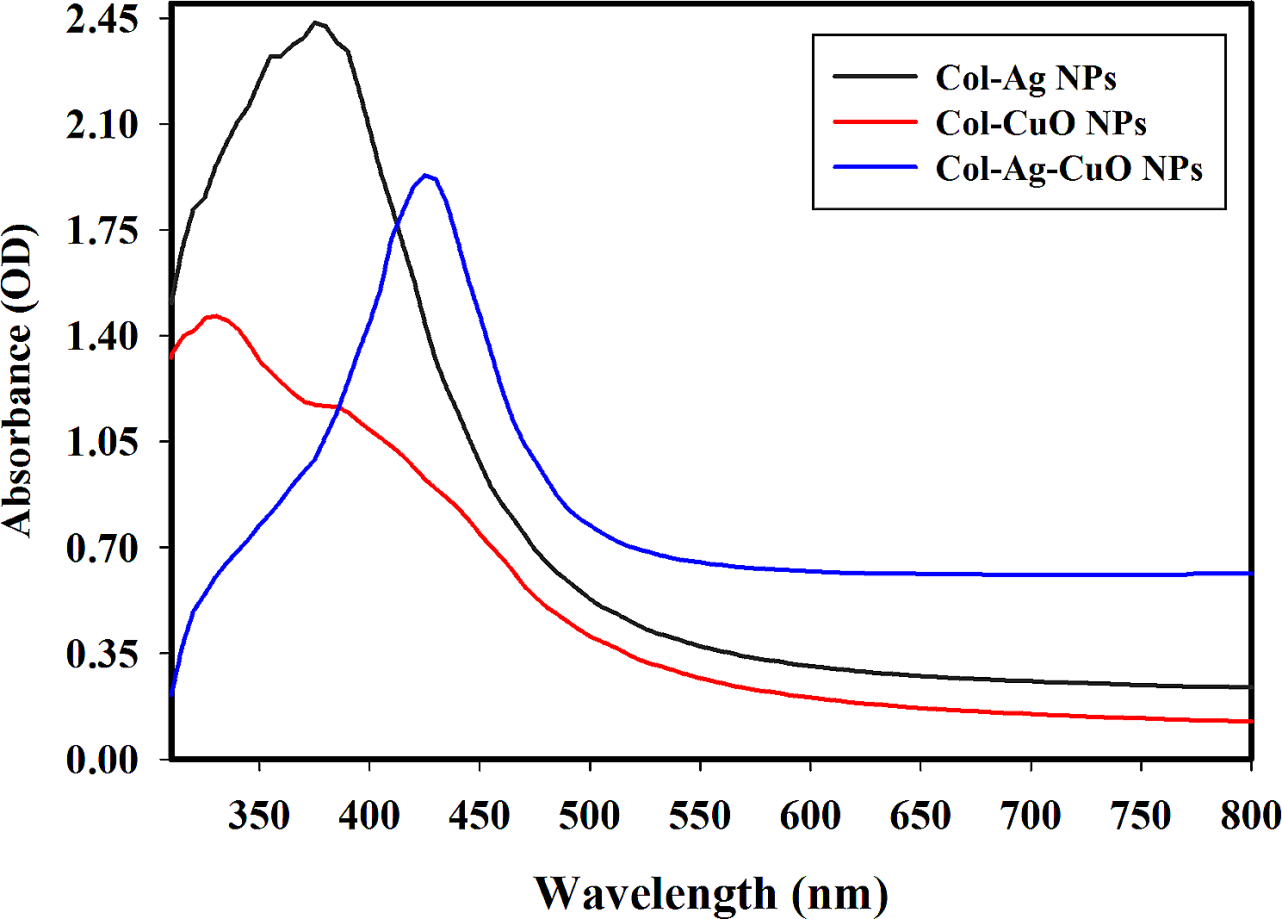
UV-Vis. spectra for the synthesized Col-Ag NPs, Col-CuO NPs and bimetallic Col-Ag-CuO NPs by 15 kGy gamma rays

Gamma-rays screening of the produced Col-Ag NPs, Col-CuO NPs and Col-Ag-CuO NPs using a UV-Vis. spectrophotometer is shown in Fig. 1. According to Fig. 1, the dosage of 15.0 kGy that produces Ag NPs with the greatest OD. = 2.4 (diluted a total of two times) at 375.0 nm is the most efficient used for their synthesis. In a similar vein, Fig. 1 demonstrates that the optimal dosage for the creation of CuO NPs is 15.0 kGy gamma rays, with an OD. of 1.5 (diluted five times) at 330.0 nm. However, Fig. 1 shows that the most effective dosage for the production of bimetallic Ag-CuO NPs is 15.0 kGy, which has a high OD. of 1.9 (diluted five times) at 425.0 nm. Gamma rays were used in this instance to produce consistent NPs with a high relative yield without the need of high temperatures or a different reducing chemical agents [34, 35].

The proportional productivity of the produced NPs had reduced when the dosage was increased by more than 15 kGy, because excess free radicals and solvated electrons (created by water radiolysis) alter the pH of the solutions, target newly formed NPs (which have different charges), interact with them, and eventually form aggregated NPs, which reduce their level of intensity in the UV-Vis. spectrum, elevated radiation is not helpful in the synthesis of new nanoparticles.

It is astonishingly discovered that the surface Plasmon resonance (SPR) of bimetallic Ag-CuO NPs solutions caused them to display a rich brownish pink hue [36, 37]. The fabrication of bimetallic Ag-CuO NPs at an elevated relative yield with low Ag and high Cu content demonstrates the current data’s scientific viability by avoiding the hazardous effects of Ag NPs in the generated bimetallic Ag-CuO NPs. Silver nanoparticles (Ag NPs) were reported in the literature to have high antimicrobial activity. However, at low concentrations, Ag NPs were found to be toxic, but when combined with copper nanoparticles (CuO NPs) to form what is known as bimetallic NPs the toxicity and the dangerous effect will be reduced. In this study, we attempted to lower the toxicity of the prepared Ag NPs by combining them with CuO NPs as one alloy as mentioned in our recent published bimetallic NPs papers [38–48].

### Characterization of Col-Ag NPs, Col-CuO NPs and Col-Ag-CuO NPs conjugates

#### SEM analysis

**Fig. 2:**
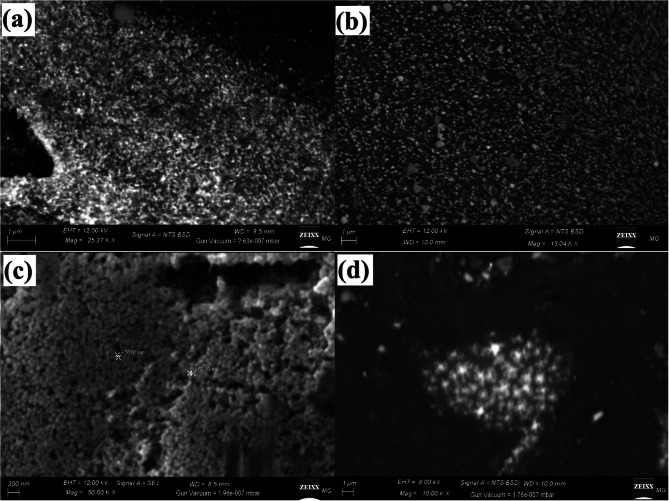
Surface morphology determination for the synthesized Col-Ag NPs **(a)**, Col-CuO NPs **(b)** and bimetallic Col-Ag-CuO NPs **(c**, and **d)** using SEM imaging

Figure 2 depicts the appearance, and surface shape of the synthesized Col-Ag NPs, Col-CuO NPs and Col-Ag-CuO NPs. Figure 2a shows that Col-Ag NPs, which appeared as brilliant particles, were evenly spread in identical form. The SEM examination of Col-CuO NPs, which likewise emerged as brilliant particles, is also shown in Fig. 2b. The SEM confirmations of the synthesized bimetallic Col-Ag-CuO NPs are shown in Fig. 2c. According to Fig. 2d, Ag and CuO NPs were dispersed uniformly. The bond formed between the colistin drug, and the synthesized NPs was intermolecular hydrogen bond as the results confirmed by FTIR analysis in recent publication [49].

The produced Ag, Cu, and Ag-CuO bimetallic NPs (in the present study) were uniformly distributed with wide size and the same spherical shape, according to a comparison of the literature about the morphological shape and analysis of elements.

Bimetallic silver and gold core-shell NPs were created by Mohsin et al. [50] using the citrate reduction process at various pH levels and temperatures. The acquired morphological form and boundary size suggested that they had a size varied from 50 to 65 nm and seem as spherical particles, which means that both pH and temperature play a crucial role in the creation process. Finally, our findings were compared with the newly published studies [51–54].

#### HR-TEM analysis

**Fig. 3:**
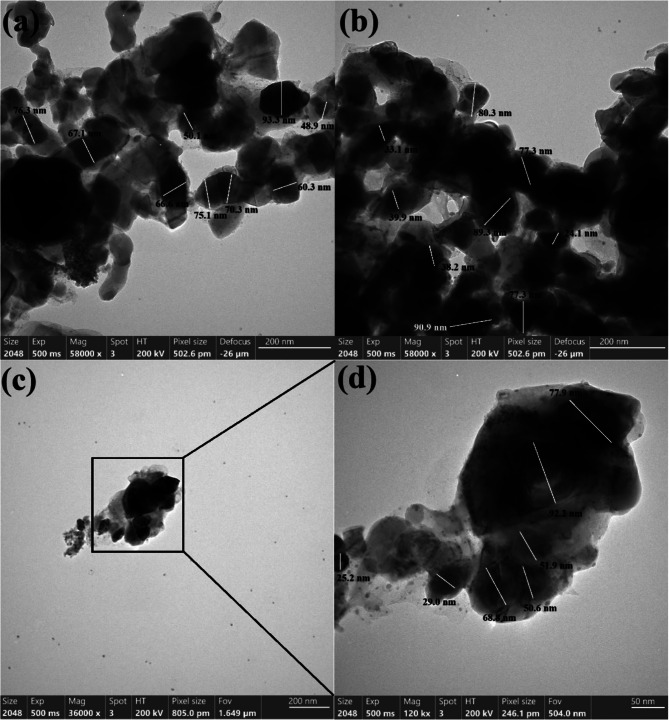
Shape, and size determination for the synthesized Col-Ag NPs **(a)**, Col-CuO NPs **(b)** and bimetallic Col-Ag-CuO NPs **(c**, and **d)** using HRTEM imaging

HR-TEM analysis was used for the characterization of shape and size of the synthesized nanoparticles (Fig. 3). It was possible to determine the mean size of the particles and see how the manufactured Col-Ag NPs, Col-CuO NPs, and bimetallic Col-Ag-CuO NPs appeared. Additionally, data from HR-TEM and DLS measurements were compared. The produced Col-Ag NPs, Col-CuO NPs, and bimetallic Col-Ag-CuO NPs have a variety of forms, including oval and spherical morphologies, as seen in HR-TEM pictures.

Col-Ag NPs varied in size from 48.9 nm to 93.3 nm, with a median diameter of 67.5 nm, as shown in Fig. 3a. Figure 3b shows the diameter range of Col-CuO NPs, which varied from 24.1 nm to 90.9 nm with a mean diameter of 68.89 nm (Fig. 3b). The dimension of bimetallic Ag-CuO NPs, on the other hand, is shown in Fig. 3c and d; it varied from 25.2 nm to 92.2 nm, with a mean diameter of 56.5 nm (Fig. 3d).

The produced Ag, CuO, and bimetallic Ag-CuO NPs varied in size and were mostly spherical in form, according to a comparison of average particle size and shape in the literature. By using the extract of a filamentous fungus, Castro-Longoria et al. [55], synthesized silver, gold, and silver-gold bimetallic. When the fungus was subjected to the water solutions of 10^− 3^ M of AgNO_3_ and HAuCl_4_, respectively, the shape of the NPs was found to be mainly spherical with a mean diameter of 11.0 nm for silver and 32.0 nm for gold.

Although different morphologies may be noticed owing to the synthetic process from extract, the anisotropic shape had been recorded, the created forms in that work [55], may be varied as the shape of extracted NPs was roughly circular or ellipsoidal in all cases. Due to the use of only one reducing and capping agent, a constant form is seen in our investigation. Finally, our findings were compared with the newly published studies [56–59].

#### DLS analysis

**Fig. 4:**
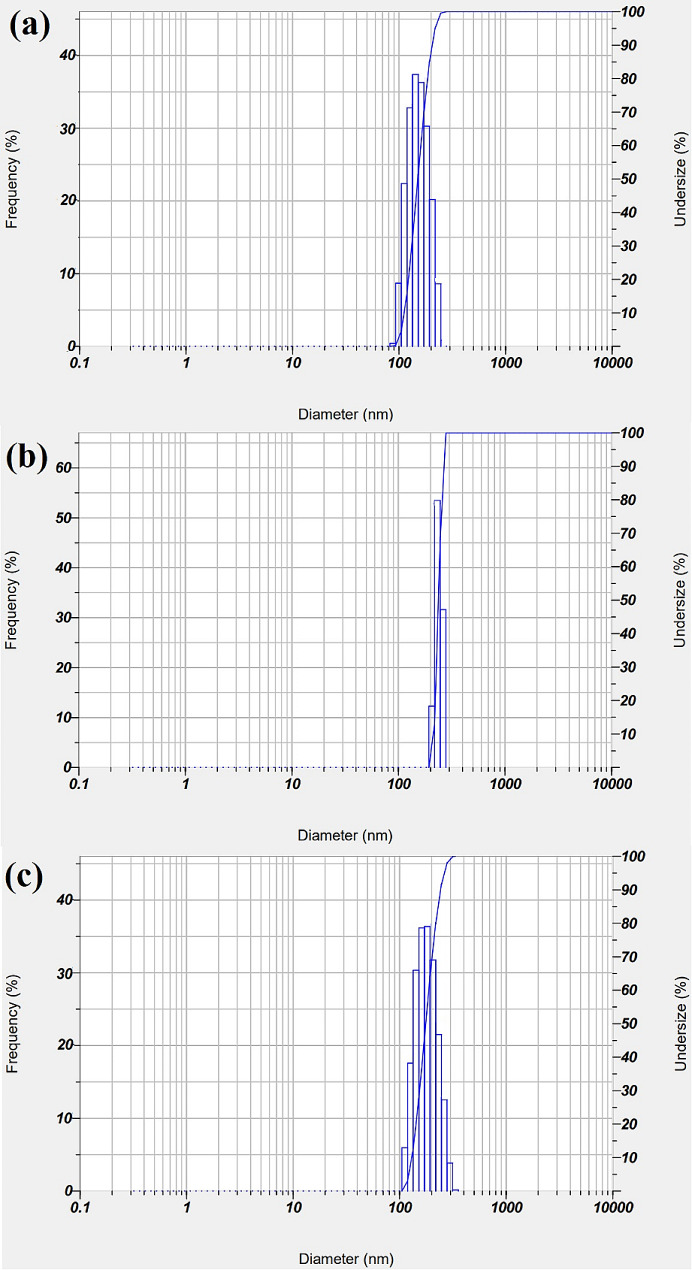
DLS distribution analysis of the synthesized **(a)** Col-Ag NPs, **(b)** Col-CuO NPs, and **(c)** bimetallic Col-Ag-CuO NPs

In order to assess the distribution of particle sizes and determine the mean size of particles for Ag NPs produced by gamma radiation in the presence of colistin, DLS analysis was carried out to find out particle size distribution. The results are shown in Fig. 4a as 181.34 nm. It was investigated to be 190.25 nm for bimetallic Ag-CuO NPs (Fig. 4c) and 189.25 nm for CuO NPs (Fig. 4b).

It is important to note that the colistin used, which served as a suitable capping and stabilizing agent, was responsible for the creation of the mono-distributed NPs [60]. As DLS analysis measures the hydrodynamic diameter of NPs bound by molecules of water (solvent), resulting in bigger sizes of the capped NPs, and HR-TEM analysis calculates the real particle size of the substance lacking solvent layer, it is common for DLS size measurements to develop greater in values compared to HR-TEM measurements [61]. The NPs that were produced were highly dispersed in a restricted range of sizes due to the scientific validity of DLS, which significantly improved their characteristics and applications [62].

#### XRD analysis

**Fig. 5:**
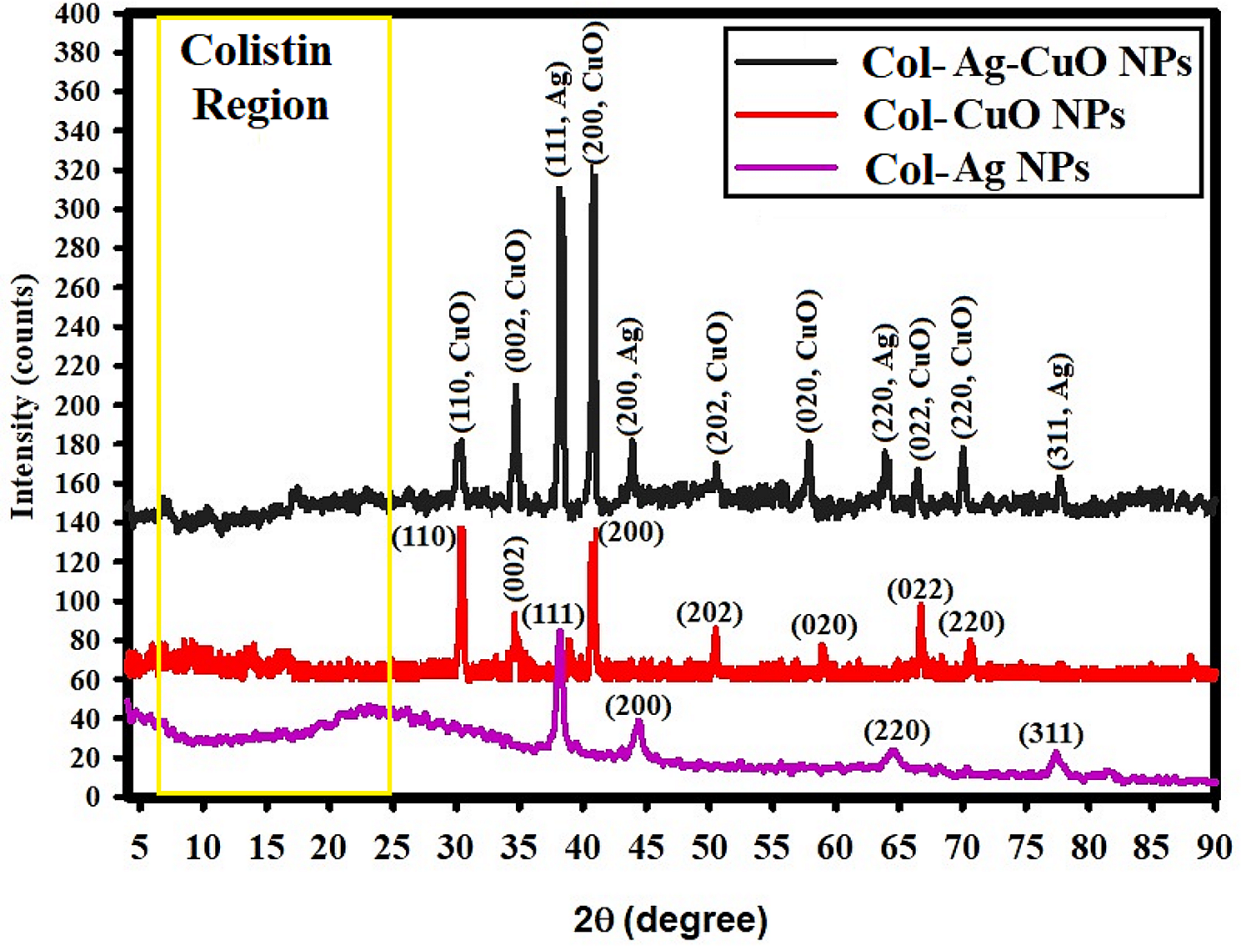
XRD analysis of the synthesized Col-Ag NPs, Col-CuO NPs, and bimetallic Col-Ag-CuO NPs

The XRD studies for the generated NPs are shown in Fig. 5. The amorphous and crystal orientations in the produced NPs, respectively, indicate which is a precursor and the produced Col-Ag NPs, Col-CuO NPs, and Col-Ag-CuO NPs. The XRD diffraction peaks of Col-Ag NPs were displayed in Fig. 5, including peaks at 2Ɵ = 38.22^o^, 44.18^o^, 63.49^o^, and 78.25^o^ that were associated with the typical card JCPDS-ICDD number 04-0783 and resembled (111), (200), (220), and (311) Bragg’s reflections [13, 63], The XRD of the synthesized Col-CuO NPs, however, revealed unique peaks at 2Ɵ = 31.23^o^, 36.81^o^, 39.89^o^, 51.56^o^, 59.86^o^, 67.12^o^, and 70.18^o^ that were accompanied by a distinctive card JCPDS number 892,531 and related to Bragg’s reflections (110), (002), (200), (202), (020), (022), and (220) [64].

Additionally, Fig. 5 displays the XRD findings of the produced bimetallic Col-Ag-CuO NPs and emphasizes the XRD diffraction peaks of Ag NPs, such as peaks at 2Ɵ = 38.22^o^, 44.18^o^, 63.49^o^, and 78.25^o^, that are accompanied by a standard card JCPDS number 361,451, and match the (111), (200), (220), and (311) Bragg’s reflections [65].

The angles (110), (002), (200), (202), (020), (022), and (220) of Bragg’s reflections are represented by the Col-CuO NP diffraction peaks at 2Ɵ = 30.19^o^, 36.19^o^, 40.12^o^, 52.09^o^, 58.40^o^, 67.40^o^, and 71.19^o^, that are supplemented by the standard card JCPDS number 892,531 [64].

The produced Col-Ag-CuO NPs were crystallized and possessed a face-centered (fcc) crystalline structure, according to the available XRD data (Fig. 5). According to the XRD data, the produced bimetallic NPs were extremely crystalline and associated with amorphous colistin , which increased their movement in the solution to enhance application [66].

The middle crystallite size of Ag NPs, CuO NPs, and bimetallic Col-Ag-CuO NPs were defined using the equation of Williamson-Hall (WH) [67, 68], and the values supplied to Eq. 1 were discovered to be 38.4 nm, 39.5 nm, and 34.7 nm, respectively.

1 $$\beta \text{cos}\theta =\frac{k\lambda }{{D}_{W-H}}+4\epsilon \text{sin}\theta$$

### Antimicrobial susceptibility

The *P. aeruginosa* isolate utilized in this study demonstrated complete resistance (100%) to all tested antimicrobial classes. Moreover, the isolate exhibited non-susceptibility to colistin, with a minimum inhibitory concentration (MIC) of 8 µg/mL. The susceptibility screening results unequivocally indicate that our *P. aeruginosa* isolate is pan-drug-resistant, rendering it non-susceptible to all antibiotics across various antimicrobial categories [69]. *P. aeruginosa* ranks among the foremost pathogens responsible for healthcare-associated infections. The emergence of pandrug- resistance in problematic Gram-negative pathogens like *P. aeruginosa* among hospitalized patients presents a significant public health threat both globally and specifically in Egypt. Yearly reports highlight increasing rates of drug-resistant bacteria [70], and in Egypt, escalating rates of resistance have been documented and linked to the indiscriminate use of antibiotics in hospital settings [4, 5].

### In-vitro antimicrobial activity of Col-Ag NPs, and Col-Ag-CuO NPs

**Fig. 6:**
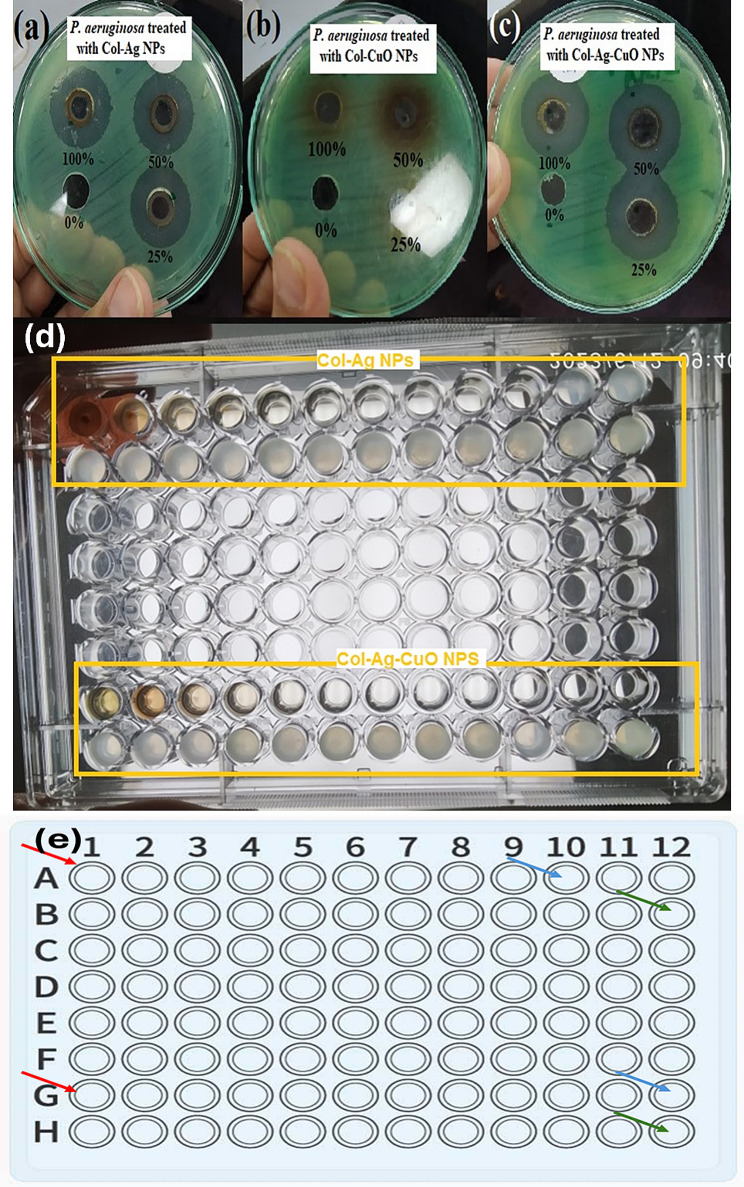
**(a, b** and **c)**. Agar well diffusion assay was performed to evaluate the antimicrobial activity of the three nanocomposite solutions at varying concentrations. **(d)**. Minimum Inhibitory Concentration (MIC) using broth microdilution method. **(e)**. Illustration panel for the MIC; rows A&B representing the dilution range of Col-Ag NPs, while **G** &**H** representing the dilution range of Col-Ag-CuO NPS, red arrows pointing to the highest concentration of the alloy (25 µg/ml), green arrows pointing to the lowest concentration of the alloys (3 × 10^− 6^ µg/ml), blue arrows pointing to the MIC

Our findings indicate that both preparations exhibited clear zones of inhibition with minimal influence of concentration on the zone diameter. However, the zones produced by the Col-Ag-CuO NPs were generally larger, as depicted in Fig. 6.

A significant reduction in the MIC of colistin was observed in both preparations compared to colistin alone. The MIC values were 8 µg/mL for colistin alone, 0.046 µg/mL for Col-Ag NPs, and 0.0117 µg/mL for Col-Ag-CuO NPs. Additionally, there was a fourfold reduction in the MIC of bimetallic Col-Ag-CuO NPs compared to Col-Ag NPs, as shown in Fig. 6. Previous studies have reported higher activity of Ag NPs against *P. aeruginosa* when conjugated with colistin compared to Ag NPs alone [1, 71]. Similarly, antibacterial activity of CuO NPs has been documented [72]. However, to our knowledge, there have been no previous reports on bimetallic Ag-CuO NPs alloy nanoparticles conjugated with colistin to assess their antibacterial activity against the gram-negative pathogen *P. aeruginosa*. Consequently, our results highlight the effective antibacterial activity of Col-Ag-CuO NPs. These findings underscore the importance of bimetallic nanoparticles conjugated with colistin as potential alternatives for treating problematic superbugs in critically ill hospitalized patients.

### Assessment of anti-virulent activity of Col-Ag-CuO NPs nanocomposite

#### Motility

**Fig. 7:**
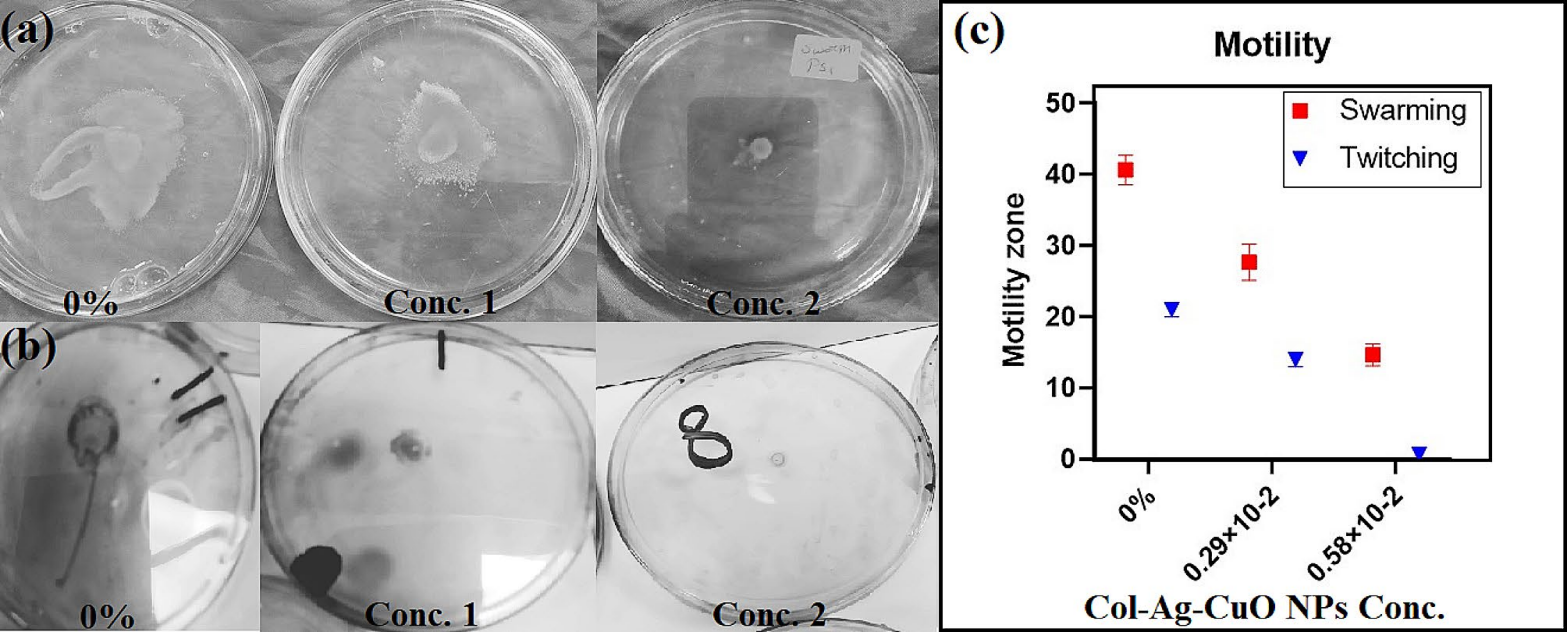
The effect of bimetallic Col-Ag-CuONPs on *P. aeruginosa* motility **(a)** reduction in swarm zone **(b)** reduction in twitching motility **(c)** The average of the 3 different readings with regard to each concentration

As shown in Fig. 7, both swarming and twitching motility were noticeably reduced with increasing nanocomposite concentration compared to the control (absence of the nanocomposite). Specifically, a reduction of 32% and 64% in the swarm zone was observed with concentration 1 (0.29 × 10^− 2^ µg/mL) and concentration 2 (0.58 × 10^− 2^ µg/mL), respectively. Similarly, twitching motility was reduced by 34% and 97%, respectively. Using ANOVA, a statistically significant difference was found on increasing nanocomposite concentration compared to the control. The P values were *P* < 0.0001 for swarming motility and *P* = 0.0005 for twitching motility.

The pathogenicity of *P. aeruginosa* and its capacity to establish infections and cause diseases are primarily attributed to the array of virulence factors it secretes [47, 73]. These secreted virulence factors play crucial roles in modulating the immune response and facilitating bacterial colonization [73]. In our study, we observed that the synthesized nanoparticles not only displayed antimicrobial properties against *P. aeruginosa* but also hindered its mobility.

This observation holds significance as mobility is often linked to the virulence of bacterial pathogens, including *P. aeruginosa*. Virulence factors empower bacteria to colonize and infiltrate host tissues, evade host immune responses, and induce disease. The mobility inhibition by the nanoparticles implies a potential interference with the expression or function of virulence factors associated with bacterial motility.

Moreover, the antimicrobial effect of the nanoparticles suggests their capability to directly impede bacterial growth and viability, thereby potentially influencing the expression of virulence factors. Although our study did not delve into the specific mechanisms underlying the interplay between nanoparticle activity, mobility inhibition, and virulence factor expression, it offers valuable preliminary insights into the potential multifaceted effects of the synthesized nanoparticles on *P. aeruginosa.*

Therefore, the activity of the bimetallic colistin nano-conjugation (Col-Ag-CuO NPs) against the secreted virulence factors of *P. aeruginosa* presents promising treatment options. This is particularly crucial given the formidable challenge posed by *P. aeruginosa* exceptional ability to acquire resistance to various antibiotics and undergo rapid mutations.

### Pyocyanin production

**Fig. 8:**
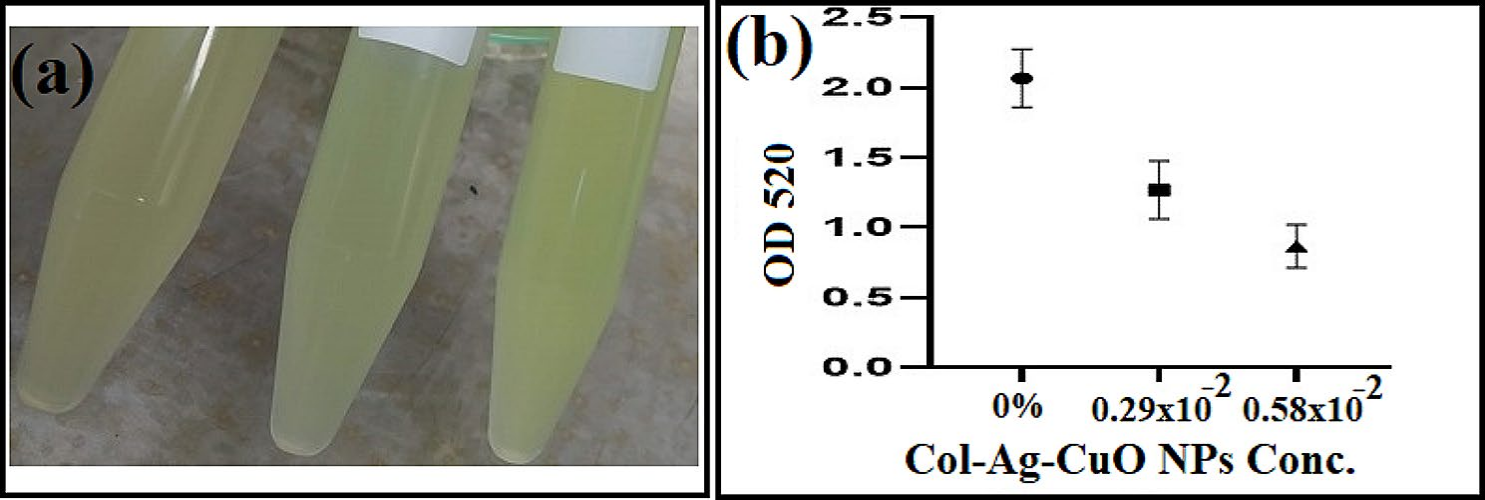
The effect of bimetallic Col-Ag-CuONPs on Pyocyanin production of *P. aeruginosa*. **(a)** Reduction in pigment production. **(b)** Means of 3 OD_520_ readings

One of the significant virulence factors of *P. aeruginosa*, contributing to its pathogenicity and severity of infections, is the production of the blue-green pigment pyocyanin [74]. Pyocyanin is known to induce tissue injury by generating reactive oxygen species [75]. The OD_520_ values of extracted pyocyanin were notably decreased following *P. aeruginosa* exposure to concentration 1 (0.29 × 10^− 2^ µg/mL) and concentration 2 (0.58 × 10^− 2^ µg/mL) of the bimetallic Col-Ag-CuO NPs nanocomposite. A reduction of 39% and 58% was observed compared to the OD_520_ value of pyocyanin extracted from the non-treated sample (Fig. 8). A statistically significant difference was observed on increasing nanocomposite concentration compared to the control. The P values was 0.0015.

### Biofilm formation

**Fig. 9:**
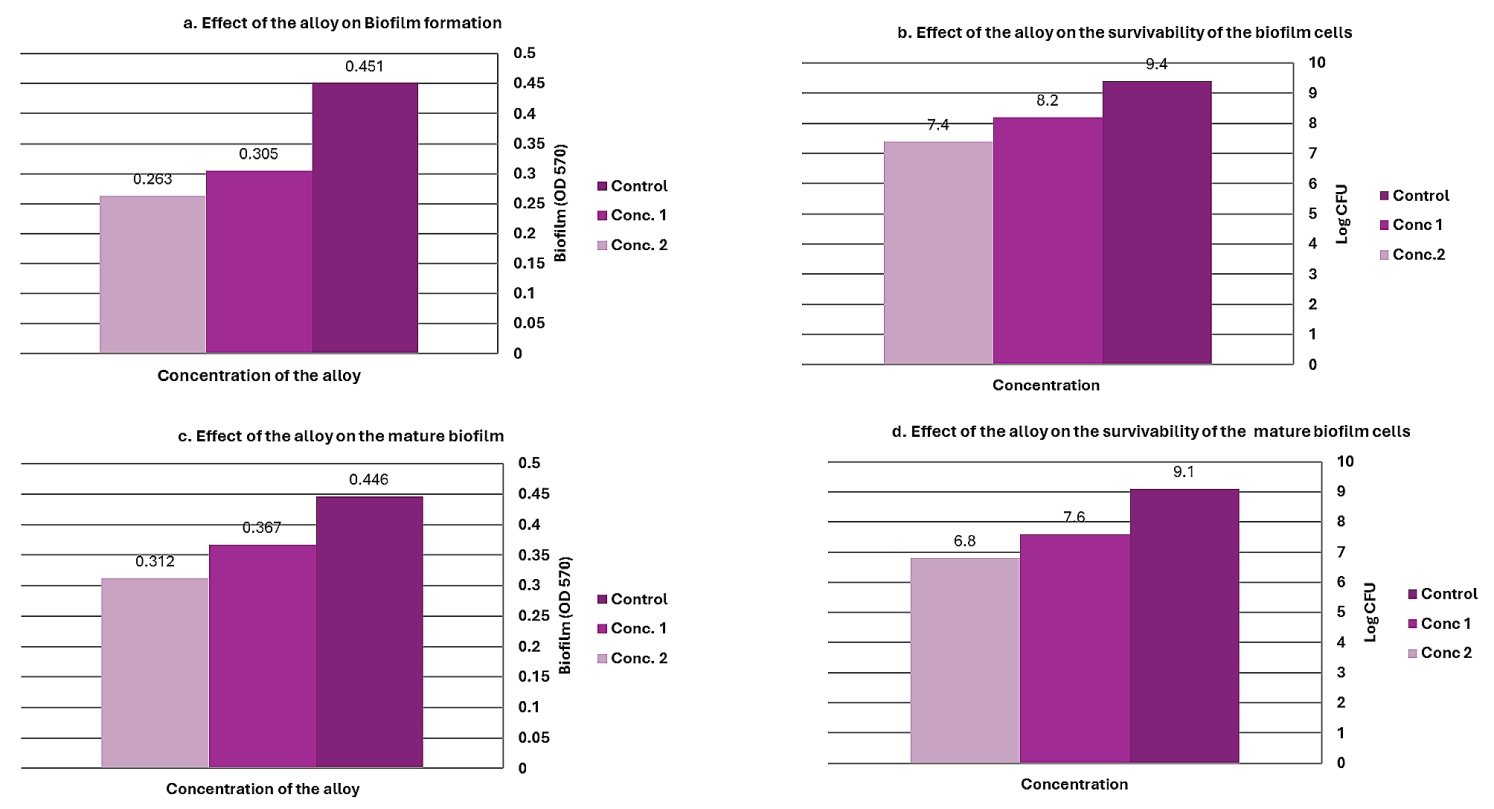
The effect of bimetallic Col-Ag-CuONPs on biofilm formation of *P. aeruginosa*

The two sub-inhibitory concentrations of the nanocomposite exhibited concentration-dependent anti-biofilm activity against our strong biofilm-producing *P. aeruginosa* isolate. Biofilm inhibition percentages were compared to the control sample at 570 nm wavelength. The higher concentration (conc 2 = 0.58 × 10^− 2^ µg/mL) demonstrated the most effective biofilm inhibition at 48%, while the lower concentration (conc 1 = 0.29 × 10^− 2^ µg/mL) reduced biofilm formation by 33%. A statistically significant difference was noticed between different treatments (*P* < 0.0001). Supportive evidence was obtained through viable counts of biofilm cells in the absence and presence of the nanocomposite. Higher concentrations decreased the viability of sessile cells by approximately 1.2 log CFU compared to the control (Fig. 9a and b). Combining results from plate-based assays and bacterial cell count assays, it can be concluded that the nanocomposite inhibited *P. aeruginosa* biofilm formation.

Eradication of mature biofilm was also observed when challenged with the bimetallic Col-Ag-CuO NPs nanocomposite compared to the control. This eradication occurred in a concentration-dependent manner: 30% and 17.7% for the higher and lower concentrations, respectively. A statistically significant difference was noticed between different treatments (*P* = 0.0037).

This is further supported by colony counts of biofilm cells, which were reduced by approximately 2.3 log CFU and 1.5 log CFU (Fig. 9c and d).

One of the main challenges in treating *P. aeruginosa* infections is the complex structure of its biofilm, which enables it to evade the immune system, making infections difficult to treat [76]. Various studies have assessed the activity of Ag NPs in hindering the biofilm formation ability of gram-negative pathogens such as *A. baumannii*, *P. aeruginosa* (synergy with tobramycin), *E. coli*, and *K. pneumoniae* [77–80]. However, this study represents the first report of decreasing biofilm activity in a strong biofilm producer *P. aeruginosa* clinical isolate using Col-Ag-CuO nano-preparation.

### In vivo activity of Col-Ag-CuO NPs nanocomposite using *G. mellonella* model

Few studies have described the toxicity of NP-antimicrobials with controversies. However, despite several studies, the current available information is insufficient to ascertain the adverse effects of NP-antimicrobials on human health. Therefore, it is imperative that further research be carried out to determine the safety profile of NP-antimicrobials to mitigate any toxicological problems that may arise.

**Fig. 10:**
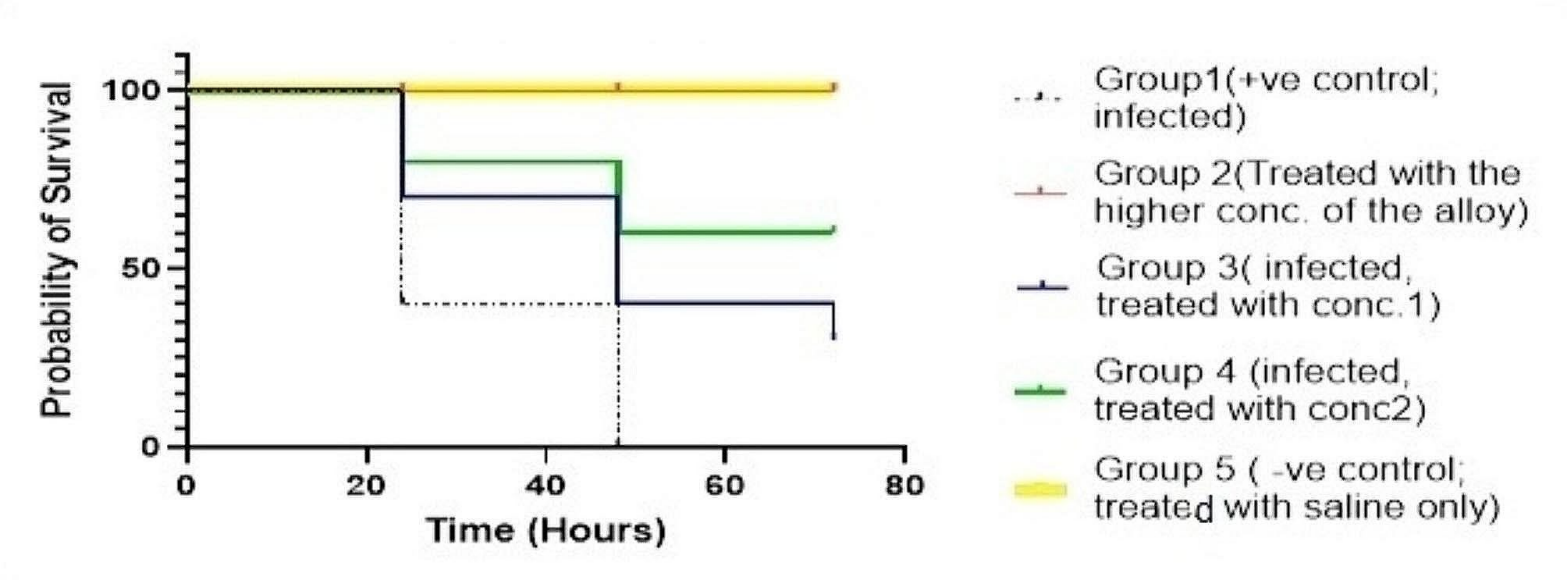
Kaplan Meier survival curve showing the effect of bimetallic Col-Ag-CuO NPs on survival of *G. melonella* infected with *P. aeruginosa*, where Group 1 is positive control; infected, Group 2 is treated with high concentration of Col-Ag-CuO NPs, Group 3 is infected and treated with concentration 1, Group 4 is infected and treated with concentration 2, Group 5 is negative control; treated with saline only

In this study, the cytotoxic effect of the Ag-CuO NPs -based colistin combination against *G. mellonella* model was evaluated to gain some insight into the safety of the tested combination. The results indicate that the nanocomposite is non-toxic for *G. mellonella*, showing similar survival rates as the saline-treated group (100% survival by day 3). Additionally, there was observed attenuation of the virulence of the *P. aeruginosa* isolate challenged with the nanocomposite compared to the control group. This attenuation occurred in a concentration-dependent manner, as demonstrated in the survival curve (Fig. 10).

The *Galleria mellonella* in vivo model offers technical, cost and ethical advantages for evaluating novel antimicrobial agents relative to mammalian models [81]. The combination of high virulence and pan-drug resistance in the isolate against commonly used antimicrobials could significantly impact morbidity and mortality rates associated with *P. aeruginosa* infections.

The findings of this study reveal that the isolate recovered from the blood of a pediatric hospitalized cancer patient is highly virulent and pan-drug resistant, posing a significant threat to patients and hospital settings while complicating therapeutic options. The data presented will require further studies before effective ‘treatment options’ can be developed.

### *Pseudomonas aeruginosa* protein leakage investigation

**Fig. 11:**
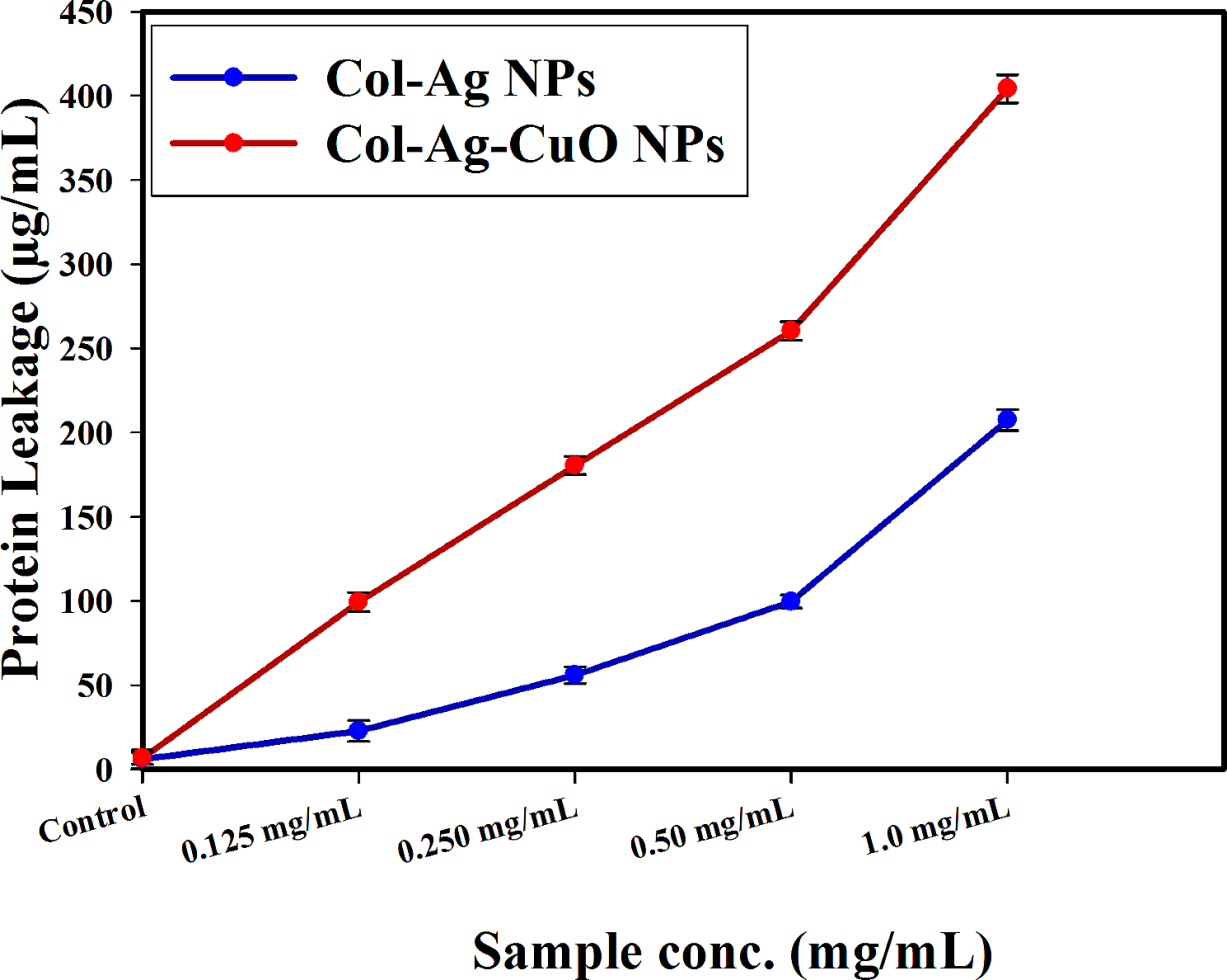
The effect of Col-Ag NPs, and bimetallic Col-Ag-CuO NPs on the protein leakage from *P. aeruginosa* cell membranes

The amount of *P. aeruginosa* protein removed (assumably as a result of pore formation which helped in making the proteins bleed out from the P. aeruginosa cytoplasm) is directly proportional to the concentration of Col-Ag NPs, and bimetallic Col-Ag-CuO NPs; it was estimated to be 207.44 µg/mL, and 404.23 µg/mL following the treatment with Col-Ag NPs and bimetallic Col-Ag-CuO NPs, respectively (1.0 mg/mL) (Fig. 11). As damage caused by the NPs progresses overtime, the leakage of cellular constituents induces cell collapse, further scoring the importance of this assay in determining cell integrity.

Similar results were reported in recent publications [82, 83]; combined NPs showed concentration-dependence for the dislodgement in the bacterial membrane and suggested leakage of bacterial intracellular organelles into the extracellular cell structure.

Ag NPs have a multi-level mode of action influencing many bacterial structures and metabolic processes including inactivating bacterial enzymes [84], disrupting cell wall, metabolic processes [85], and increasing cell permeability [86, 87]. Ag NPs can also interact with DNA [84], or generate reactive oxygen species [88], which damage biomacromolecules [89].

Nanomolecules (NM) act along two major lethal pathways, which are related to each other and in many cases occur simultaneously. The antimicrobial properties of Ag NPs can be attributed to multiple different mechanisms, including: (i) disruption of cell membrane integrity resulting in pore formation in treated bacterial cells leading to an increase in permeability allowing antibiotics to enter the cells more easily, and (ii) production of reactive oxygen-free radicals with the NM acting as nano-catalysts.

Figure 11 shows the effect of Col-Ag NPs and bimetallic Col-Ag-CuO NPs on the protein leakage from *P. aeruginosa* cell membrane. Protein leakage assay and/or SEM imaging are used for evaluating the antimicrobial potential of synthesized nanoparticles [39]. In some studies, SEM imaging process was used to confirm the pore formation in the treated bacterial cells [39, 90, 91]. In this study, the protein leakage assay was used to assess *P. aeruginosa* integrity following treatment with NPs in comparison with non-treated control.

## Conclusion

This study investigated the use of bimetallic silver-copper oxide nanoparticles (Ag-CuO NPs) conjugated with colistin to combat a highly virulent, pandrug-resistant clinical isolate of *P. aeruginosa.* The combination significantly lowered the minimum inhibitory concentration (MIC) of colistin and decreased swarming and twitching motility, pyocyanin production, and biofilm formation of *P. aeruginosa.* Additionally, the nanoparticles were non-toxic to *Galleria mellonella*, showing 100% survival, comparable to saline-treated controls. This research offers a promising approach to tackle antibiotic-resistant *P. aeruginosa* strains, including those resistant to colistin. By leveraging the synergistic effects of silver and copper oxide nanoparticles, the study presents a novel method to combat resistant infections through mechanisms such as reactive oxygen species generation and membrane disruption. The positive results suggest that colistin-conjugated Ag-CuO NPs could be developed for treating difficult infections, particularly in immunocompromised patients. Furthermore, the findings could inform nanoparticle-based therapies against other multidrug-resistant pathogens, aiding global efforts to combat antimicrobial resistance. However further research is needed to consolidate the reported findings.

## Data Availability

The datasets used and/or analyzed during the current study are available from the corresponding author on reasonable request.
